# Thin HPFRC Jackets for Axially Loaded RC Columns: Mechanical Behavior and Efficacy of Strengthening

**DOI:** 10.3390/ma19020220

**Published:** 2026-01-06

**Authors:** Maria Dolores Criado Fernández, Sonia Martínez de Mingo, Ana Almerich-Chulia

**Affiliations:** 1 Doctoral School, Universitat Politècnica de València, 46022 Valencia, Spain; mariadolores.criado@ietcc.csic.es; 2 T.U. Experimental Evaluation of Structures, Eduardo Torroja Institute of Construction Sciences (IETCC), CSIC, 28033 Madrid, Spain; soniamdm@ietcc.csic.es; 3 Department of Continuum Mechanics and Structural Theory, Universitat Politècnica de València, 46022 Valencia, Spain

**Keywords:** high-performance fiber-reinforced concrete, RC columns, structural strengthening, jacketing, sustainability, axial compression, load-bearing capacity

## Abstract

The environmental impact of the construction sector underscores the urgent need for sustainable solutions to extend the service life of existing structures. This study explores High-Performance Fiber-Reinforced Concrete (HPFRC) for strengthening reinforced-concrete (RC) columns subjected to axial compression. Twelve RC columns were tested, each 1200 mm high and with varying cross-sectional shapes (circular, square, and rectangular). Strengthening was achieved using thin HPFRC jackets (less than 30 mm thick), applied without additional internal reinforcement and following simple surface preparation techniques such as sandblasting. Full-height jacketing significantly improved axial load capacity. Its effectiveness did not decrease with the shape of the cross-section, with square columns showing up to a 105% increase and rectangular ones up to 87%, compared to unstrengthened columns with the same concrete strength. The highest improvement was observed in the square column with full-height jacketing and the most significant geometric strengthening ratio (52.6%), which doubled its axial capacity. This ratio was directly related to performance gains. Although ductility gains were limited, the full-jacketed specimens did not fail explosively: their failure mode was progressive, providing a useful warning before collapse. HPFRC jacketing can be especially effective for non-circular columns, outperforming FRP jacketing and eliminating the need for additional protective layers against impact or fire.

## 1. Introduction

Reinforced concrete (RC) is undoubtedly the most widely used material in the construction of modern infrastructures and buildings due to its good mechanical strength, versatility, ease of execution, and relatively low cost. However, much of the existing structural stock has deteriorated and requires repair or modernization due to durability problems, carbonation phenomena, chloride penetration, corrosion of the reinforcement, and exposure to aggressive environments. On top of this is the natural aging of structures, damage following an earthquake, or increases in the design load due to changes and updates in codes and design standards, as well as functional changes and/or construction errors [1,2,3], which make the reinforcement, rehabilitation, and restoration of RC pillars and columns necessary [4].

It is essential to leverage new materials and emerging technologies in structural rehabilitation and reinforcement to extend the lifespan of buildings more sustainably, with a lower environmental impact being achieved by reducing material consumption, increasing service life, and reducing maintenance.

Reinforcement techniques range from traditional ones, such as conventional concrete cover [5,6], the use of fiber reinforced polymers (FRPs) [7,8] and metal jackets [9,10], to the most innovative ones, such as engineering cementitious composites (ECCs) and epoxy injection and grouting [11,12]. Although these solutions have proven effective in many cases, they also have certain disadvantages, such as increased structural weight, corrosion and fire resistance problems, adhesion difficulties, or the degradation of the adhesive materials used on the interaction face of the FRP [4,13]. Due to advances in materials, work is being performed to find new, efficient covering techniques capable of improving structural performance, with minimal increases in dimensions, where a smaller amount of material is used and with superior durability [14,15].

**Figure 1:**
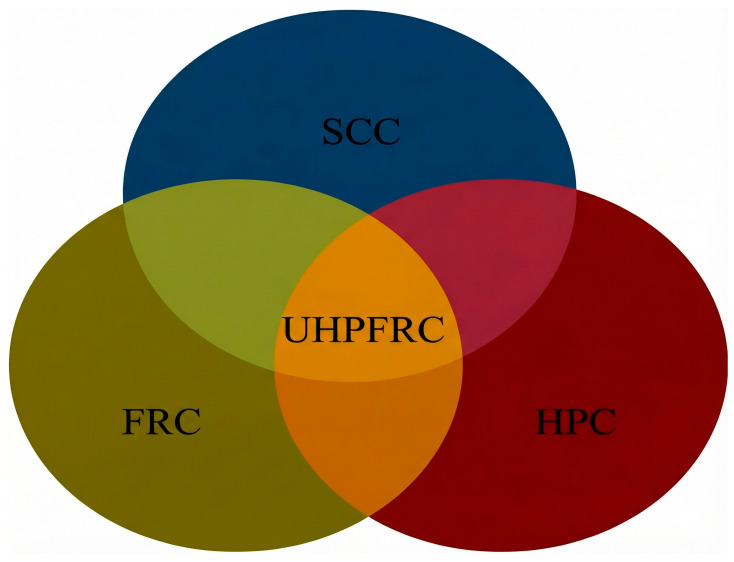
Types of special concrete [16,17].

Recent advances in the development of cement, the introduction of fibers for structural reinforcement, and significant progress in water-reducing admixtures have resulted in various types of specialty concrete. One of these is High-Performance Fiber-Reinforced Concrete (HPFRC), which incorporates advances in Self-Compacting Concrete (SCC), Fiber-Reinforced Concrete (FRC), and High-Performance Concrete (HPC) technologies, as shown in Figure 1 [16,17]. HPFRC is produced from cement, water, additives and short steel fibers; fine aggregates and various chemical additives are occasionally used. Different combinations of these materials can be found, giving rise to the different families of UHPC-HPFRC, referring to concretes that combine the concepts of Ultra-High-Performance Concrete and Fiber-Reinforced Concrete, depending on the application and the supplier [16,17,18,19].

Due to the absence of coarse aggregates in the patented mixtures, some researchers define HPFRC as not being a conventional concrete. For this reason, various research projects use the term “mortar” or High-Performance Fiber-Reinforced Mortar (HPFRM), and others use the term “reactive powder concrete” (RPC) [17].

HPFRC is characterized by its high fluidity, initial strength, ultimate strength, and superior durability. Its mechanical properties far exceed those of conventional concrete, depending on its composition, and include the following: compressive strengths starting from 100 to 150 MPa; high post-cracking tensile strength, due to the presence of metallic fibers, of up to 7–10 MPa; ultimate compressive strains greater than 1%; very dense microstructures (better durability); and the ability to control cracking and dissipate energy under cyclic/seismic loading [20,21]. Furthermore, HPFRC exhibits an adhesion to conventional concrete greater than 20 MPa, high toughness, and practically zero chloride permeability, resulting in exceptional durability. These characteristics make it an ideal material for new structures and rehabilitation using thin jackets (20–50 mm) [22], minimizing the architectural and functional impact.

Research on the use of fiber-reinforced HPC for strengthening RC columns has grown significantly over the last 15 years. Numerous experimental and analytical studies have been conducted to evaluate the behavior of RC columns reinforced with HPFRC jackets.

Lampropoulos et al. carried out one of the pioneering studies on HPFRC jackets in columns, analyzing the effect of parameters such as jacket thickness, concrete shrinkage, and the addition of steel bars, highlighting improvements in strength and ductility [23]. Cassese et al., Dadvar et al., and Shehab et al. tested RC columns strengthened with an HPFRC jacket without additional reinforcement. The results showed a significant improvement in the structural performance of the strengthened columns [20,21,24]. Similarly, Elsayed et al., Wang et al. and Xie et al. [25,26,27] analyzed the reinforcement thickness parameter and specimen shapes, demonstrating that the jackets increased both the bearing capacity and stiffness. Studies by Le et al. on circular columns, Susilorini and Kusumawardaningsih on square columns, and Alamoodi et al. on circular, square and rectangular columns confirm the achievement of increases in the axial capacity of jacketed columns with thicknesses of 20–40 mm [22,28,29].

Cho et al. and Li et al. [30,31] provide further evidence on the effectiveness of HPFRC in improving axial stiffness, while Shin et al. [32] highlight the confinement capacity provided by this material. Similarly, Gholampour et al. [33] combined experimental tests and numerical simulations to demonstrate that HPFRC reinforcement increases strength, reduces deformations, and improves ductility. More recent studies, such as those by Tolentino et al. [34], have consolidated these results, confirming that thin HPFRC jackets can be highly effective in the rehabilitation of columns subjected to axial compression.

Shehab et al. [21] analyzed the effect of surface preparation, confirming that techniques such as vertical grooving improve the adhesion between the substrate and the jacket. Braveru et al. [35] evaluated square columns with HPFRC jackets, highlighting similar improvements and underlining the importance of core–jacket adhesion.

HPFRC has shown great potential for rehabilitating columns. Meda et al. and Betar et al. investigated the repair and reinforcement of damaged reinforced-concrete columns with an HPFRC jacket and verified that it can restore the initial strength [36,37]. From their experimental results, they observed an improvement in column strength and durability, in addition to maintaining almost the same geometry of the original structure, due to the reduced thickness of the jacket; Chen et al. [38] applied HPFRC jackets to corroded columns, managing to restore their capacity and protect the reinforcements against further corrosion. Tolentino et al. [34] demonstrated that even in severely damaged columns, the application of HPFRC jackets significantly increases stiffness and strength, extending the service life of the structures.

Likewise, there are studies on the behavior of HPFRC jackets in reinforced-concrete columns under cyclic loading and seismic behavior. Hong et al. [39] demonstrated that reinforcement with HPFRC under cyclic lateral loading increases ductility and energy dissipation, reducing accumulated damage. Gu and Zhang [40] carried out a parametric study of jacket thickness, demonstrating that thicknesses between 25 and 35 mm achieve an optimal balance between stiffness, ductility, and cost. Shao et al. [4] performed full-scale column tests with high axial load demands, using both in situ and prefabricated UHPC jackets. Beschi et al. [41] demonstrated in their research that reinforcement with HPFRC (40 mm) for the static and seismic loading of reinforced-concrete columns represents a practical alternative solution.

Reviews such as those by Saeed and Hejazi, Niu et al., and Chitty et al. summarize the progress achieved, demonstrating that HPFRC jacketing is an effective technique for the rehabilitation of RC columns, providing increases in bearing capacity, stiffness and ductility, as well as long-lasting corrosion protection. Its main advantages over traditional techniques include its reduced thickness and the material’s excellent durability. However, they also highlight the need for optimizing thickness and the surface treatments used, as well as more the need for full-scale tests and durability studies [13,42,43].

These factors provided the motivation for the present experimental work, which aims to provide additional evidence and new applied perspectives, addressing some of the gaps identified in the scientific literature. This study focuses on applying a thin HPFRC jacket to real-life cases of column reinforcement, primarily in the construction sector, through minimal intervention, as an effective solution for centered compression strengthening.

A review of the scientific literature on HPFRC jackets without additional reinforcement for axial compression strengthening reveals a lack of experimental studies on medium- or full-scale specimens. Most existing tests have been conducted on small-scale columns, with heights typically between 300 and 700 mm [20,26,27,29], as well as heights reaching 1000 mm in rare cases [21]. Moreover, the cross-sections of these specimens are usually small (diameters or sides between 120 and 150 mm [20,21,27]), leading to unrealistic ratios between the thickness of the jacket (15–40 mm) and the size of the section. This limitation prevents a proper assessment of the technique’s performance under realistic structural conditions.

Therefore, the present experimental program was designed to evaluate the effectiveness of this strengthening technique on medium-scale RC columns (1200 mm high) under axial compression, using thin HPFRC jackets without additional vertical or transverse reinforcement. This study aims to provide data from specimens with dimensions closer to those of real RC columns, considering variables such as jacket height, section geometry, and the geometric strengthening ratio, thus contributing to a more representative understanding of the behavior of HPFRC jacketing.

## 2. Materials and Methods

### 2.1. Experimental Program

The study aims to evaluate the efficacy of the strengthening using HPFRC jackets applied to the kind of columns commonly used in building construction, so that their axial compressive capacity is improved through minimal intervention. For this reason, the experimental program seeks to replicate the actual conditions of columns in buildings: concrete strength around 25–30 MPa, moderate internal steel reinforcement, typical cross-sectional dimensions, vertical casting of both the concrete column and the HPFRC jacket, substrate preparation limited to sandblasting, jackets without additional internal reinforcement, etc.

The main variables of the study are:

- The shape of the cross-section: circular, square, and rectangular with varying side ratios (length-to-width ratios of 1.5 and 2).
- The height of the jacketing: either over the full height of the column (h_m_ = 1200 mm) or leaving the top 5 cm unjacketed (h_m_ = 1150 mm). This variable is studied only in the circular and square specimens.

Although not initially intended as variable parameters, other factors influencing the study’s results include:

- The compressive strength of the concrete may vary from one production batch to another. Its value at the time of testing (f_c_) is known, as control cylindrical specimens were tested on the same day as the reinforced column.
- The geometric strengthening ratio (ρ_m_) is similar across specimens but not identical. It is the result of the cross-section’s specific shape, the effect of sandblasting, and the dimensions of the commercial formworks used for casting the jacket.

### 2.2. Specimen Details

Twelve RC columns were tested, each 1200 mm high. Table 1 summarizes their main characteristics: total specimen height (H); height of the strengthening jacket (h_m_); diameter or width of the original cross-section (b); length of the original section when rectangular (a); thickness of the jacket measured in both directions (t_b_ and t_a_); compressive strength of the concrete (f_c_) (obtained from cylindrical specimen tests on the date of testing); area of longitudinal steel reinforcement (A_s_); concrete gross area before strengthening, excluding internal steel reinforcement (A_n_); jacket area (A_m_); and geometric strengthening ratio, defined as the ratio between the jacket’s area and the net area of the concrete before strengthening (ρ_m_ = A_m_/A_n_).

In addition, Table 1 includes the ratio between the thickness of the jacket and the size of the section of the strengthened column (t/b_ext_), where b_ext_ is the overall diameter or width of the strengthened cross-section, calculated as b_ext_ = b + 2t.

Three columns had a circular cross-section (denoted by the letter C in their name), four have a square cross-section (S), three have a rectangular cross-section with a side ratio of 1.5 (R), and two have a rectangular cross-section with a side ratio of 2 (XR). The columns were cast using reused wooden formworks with rounded corners, having a rounding radius of 20 mm.

One column of each cross-sectional type was tested without strengthening as a reference (C0, S0, R0, and XR0). The remaining columns were strengthened with an external HPFRC jacket with a height of either 1200 mm or 1150 mm. The thickness of the mortar jacket was less than 30 mm.

**Figure 2:**
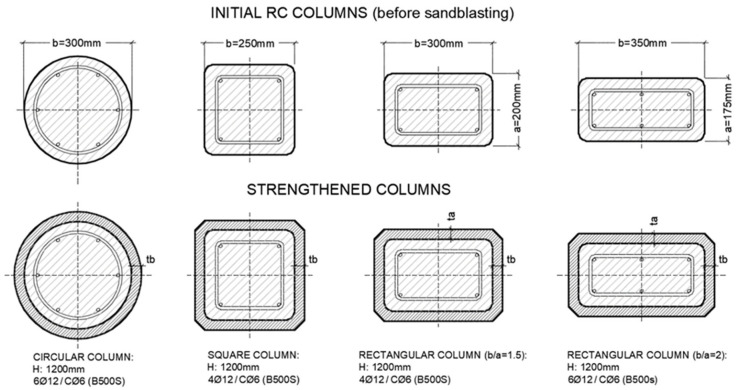
Geometric details of the initial and strengthened RC columns.

**Figure 3:**
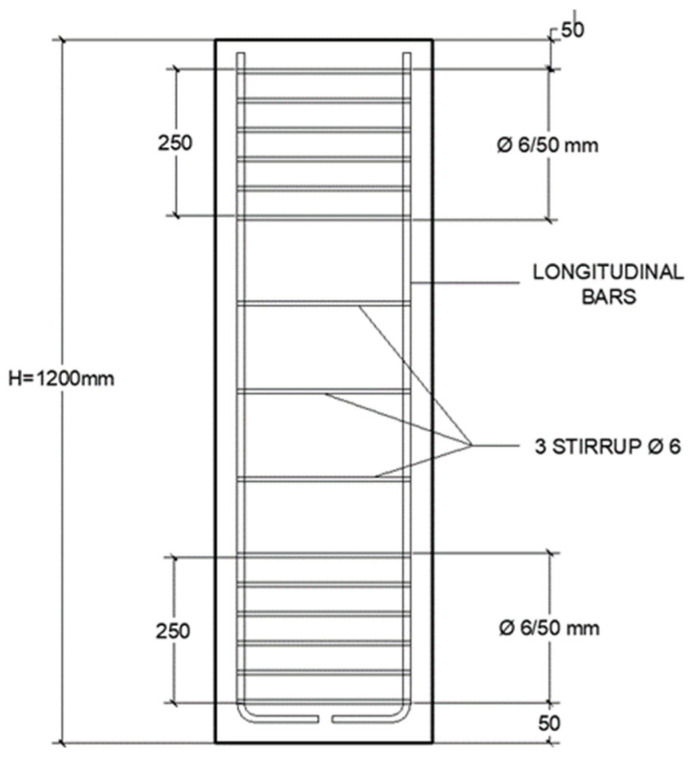
Internal steel reinforcement of columns.

The specimens had similar concrete cross-sectional areas and were reinforced with B500S steel (f_ys_ = 500 MPa, E_s_ = 200,000 MPa). Figure 2 and Figure 3 show the internal steel reinforcement. The columns were cast with concrete with a compressive strength of approximately 25–30 MPa, although higher strength was achieved in some cases.

In this study, the ratio of the thickness of the jacket to the size of the inner concrete core ranges between 7.1 and 12.5.

### 2.3. Materials for Strengthening

The jacketing was produced using a two-component cement-based mortar “Planitop HPC” by Mapei Spain, composed of high-strength cements, selected aggregates, and special additives [44]. It is reinforced with rigid, straight-ended metallic fibers, manufactured from cold-drawn wire, with an aspect ratio of 62. The fiber volume content is approximately 2%. According to the manufacturer’s data, the mechanical properties after 28 days include a compressive strength of 130 MPa and a flexural strength of 32 MPa.

When the jacket covers the entire column height, the top 50 mm are cast using a mortar with similar mechanical properties but different workability, “Planitop HPC Tixo” by Mapei Spain [45]. This denser mortar, which can be applied with a trowel, is typically used on-site to finish the upper part of the jacket. According to the manufacturer, its compressive strength at 28 days is 100 MPa.

To facilitate the placement of the mortar, a superplasticizer is used, “Mapecure SRA” by Mapei Spain [46], which, according to the product datasheet, reduces the mechanical properties by approximately 5% to 6%.

### 2.4. RC Columns Strengthened with HPFRC Jacket

Before the strengthening process, the columns were sandblasted to ensure good adhesion, slightly reducing their original cross-sectional dimensions (the resulting dimensions are listed in Table 1). Sand with a very fine particle size distribution (0.0–0.3 mm) was used for the sandblasting process to achieve a uniform surface texture.

**Figure 4:**
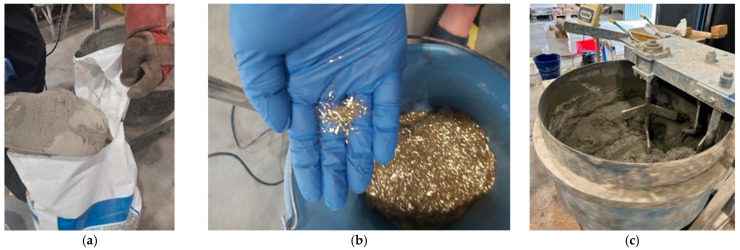
HPFRC: (**a**) Mortar, (**b**) steel fibers, and (**c**) mixing the materials.

The HPFRC material was made following the dosage and instructions provided in the product’s technical datasheet. The pre-dosed dry mortar had water and rigid steel fibers added to it (Figure 4). First, the mortar powder was mixed with the mixing water, which had been previously combined with the superplasticizer. After the mixing time specified in the datasheet, the steel fibers were gradually added manually while mixing continued until the process was completed within the required time.

**Figure 5:**
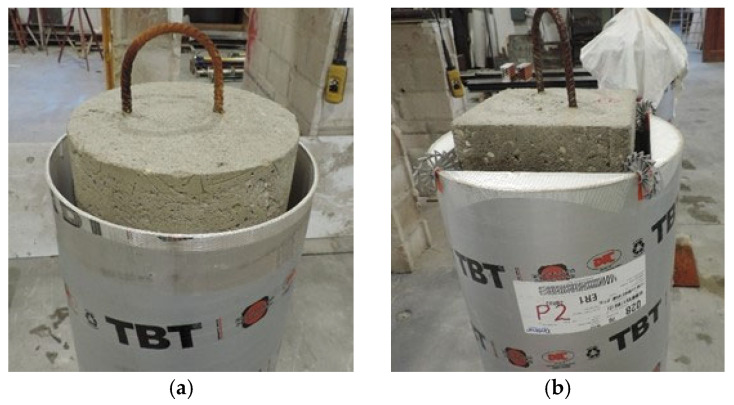
Specimens’ appearance before casting the HPFRC: (**a**) circular, (**b**) square.

While the concrete columns were cleaned of dust using compressed air and moistened, new formwork was installed to shape the jacket. In this case, commercial non-recoverable formworks were used (Figure 5).

**Figure 6:**
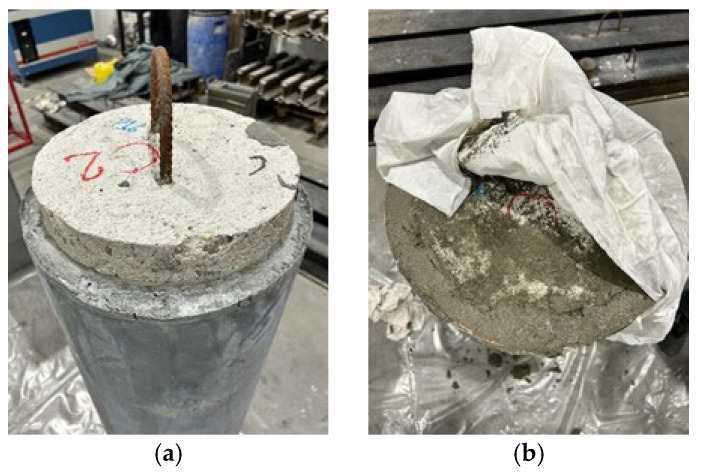
(**a**) Circular column with initial jacketing up to a height of 1150 mm; (**b**) subsequent leveling of the jacket to the top with thixotropic mortar.

Once the mixing was completed, the HPFRC was poured vertically up to an initial height of 1150 mm. This height replicates on-site conditions due to the difficulty of pouring the mortar in a single operation over the full column height. Some circular and square specimens were tested with this height (1150 mm). For the remaining specimens, the jacket was extended over the full height, with the final 50 mm topped off using a trowel-applied mortar “Planitop HPC Tixo”, thus leveling the jacket with the top surface of the specimen (Figure 6).

Proper curing of this type of mortar is essential. In this study, curing was carried out under laboratory conditions by covering the specimens and columns with wet clothes for 72 h, after which the formwork was removed.

### 2.5. Instrumentation and Test Setup

**Figure 7:**
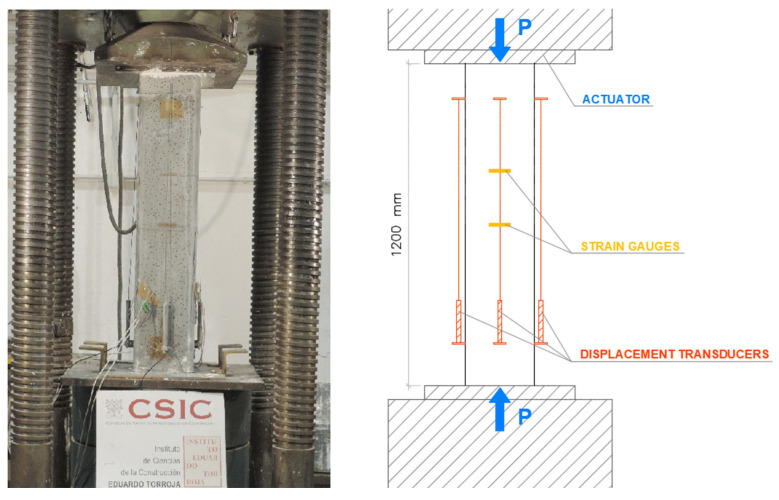
Test setup for specimen R1_h1200.

At least 28 days after the strengthening was completed, the columns were tested under centered axial compression. A 10,000 kN Icon press—class 2—was used for this purpose. Figure 7 shows the general setup of the test for one of the columns. The specimens were instrumented around their perimeter to study their mechanical behavior. Specifically, displacement transducers were used to measure axial strains, and strain gauges were used to measure both axial and lateral strains.

The axial strain was measured using displacement transducers that recorded the shortening of the column over a reference length of 1000 mm. For the circular columns, three transducers were placed at 120° intervals. For the other specimens, four transducers were positioned, one at the center of each face. A strain gauge strip was also bonded in the axial direction to monitor the shortening.

The lateral strain was measured using strain gauges bonded at the mid-height of each column (three strips at 120° for circular columns and four strips for the others).

**Figure 8:**
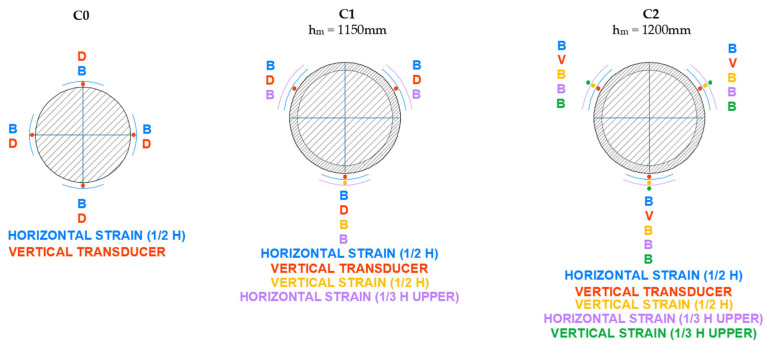
Setup of instrumentation on circular cross-section columns (C).

**Figure 9:**
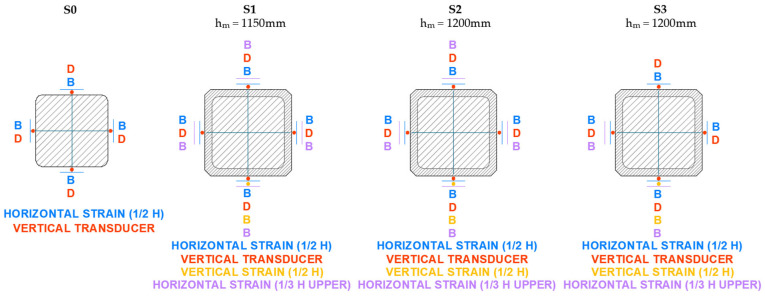
Setup of instrumentation on square cross-section columns (S).

**Figure 10:**
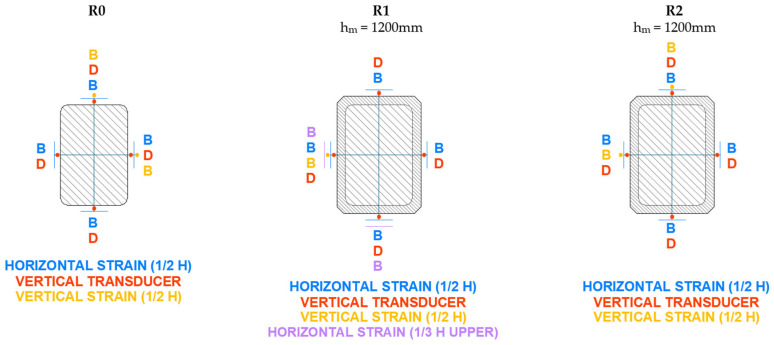
Setup of instrumentation on rectangular cross-section columns with b/a = 1.5 (R).

**Figure 11:**
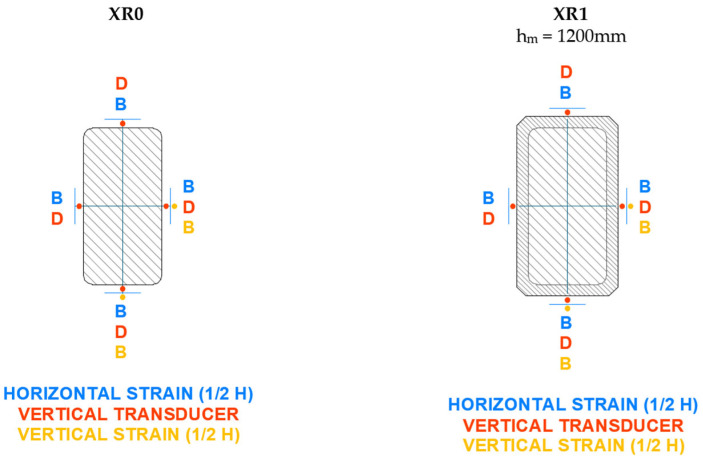
Setup of instrumentation on rectangular cross-section columns with b/a = 2 (XR).

The instrumentation layout used in the tests is shown in detail in Figure 8, Figure 9, Figure 10 and Figure 11. As can be seen, in addition to the strips placed at mid-height, the upper third of the specimen was also instrumented, although with fewer sensors. All columns were tested until failure. During the tests, readings from the transducers, strain gauges, and the applied load were continuously recorded.

## 3. Results

### 3.1. Analytical Results

Table 2 presents the main results obtained from the tests, including: the maximum load recorded during the test (P_max_), as well as the axial and lateral strains measured in the column for the maximum load (ε_c,axial_ and ε_c,lat_). The table also includes additional parameters calculated from the test results to assess the effectiveness of the strengthening.

- The average stress in the central section (f′_c_) is estimated assuming the entire section would be subjected to uniform stress. This is a simplified parameter used to compare the improvement in strength achieved through the strengthening. It was calculated as the ratio of the maximum test load (excluding the portion resisted by the longitudinal steel reinforcement) to the concrete’s cross-sectional area. The axial strain in the reinforcement at maximum load (ε_s,axial_) was assumed to be equal to the strain recorded in the specimen during the test (ε_c,axial_). Previous experimental studies have shown that the axial strain in the internal steel reinforcement is similar to that of the jacket [29].

(1) $$f\prime_{c}=[P_{max}-(A_{s}E_{s}\varepsilon_{s,axial})]/(A_{n}+A_{m})$$

- The ratio between this average stress and the strength of the concrete at testing time (f′_c_/f_c_).
- The theoretical axial load of an unstrengthened column (P_o,theo_) is calculated based on the actual compressive strength of the concrete obtained experimentally at the time of testing (f_c_) and the contribution of the longitudinal reinforcement (f_s_). The latter is estimated assuming that the steel undergoes the same strain as the concrete (0.002), which implies that its stress does not exceed 400 N/mm^2^ (f_s_ = E_s_ · 0.002).

(2) $$P_{o,theo}=f_{c}\cdot A_{n}+A_{s}\cdot f_{s}$$

- The ratio between the maximum test load and the theoretical axial load of an unstrengthened column (P_max_/P_o,theo_).

In addition, Table 2 includes the arithmetic mean (M), standard deviation (SD), and coefficient of variation (CV) for cases where several specimens were tested with the same type of cross-section and jacket height.

### 3.2. Mechanical Behavior of the Specimens

The mechanical behavior of the elements is analyzed based on axial load vs. strain curves, which are plotted using the test results.

The axial strain is estimated as the average of the readings obtained from the displacement transducers. The lateral strain is calculated as the average data collected from the strain gauge strips bonded transversely at the specimen’s central section (mid-height). Additional data from strain gauges placed in other configurations were also analyzed, confirming that the readings are consistent with those used in the final analysis and do not provide any additional information.

**Figure 12:**
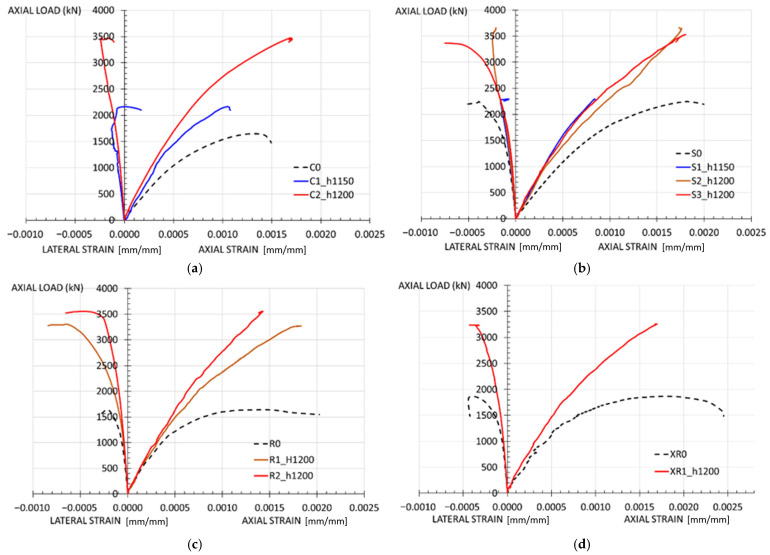
Load–strain curves for: (**a**) Circular columns; (**b**) Square columns; (**c**) Rectangular columns with b/a = 1.5; (**d**) Rectangular column with b/a = 2.

Figure 12 shows the curves obtained from the tests on columns with the same type of cross-section.

**Figure 13:**
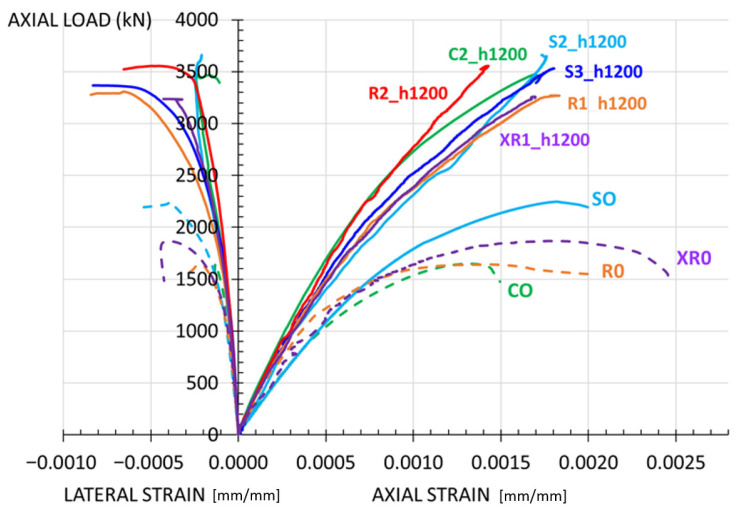
Load–strain curves for columns with full-height jacketing.

Figure 13 presents all the curves corresponding to specimens where the jacket covers the full height (h_m_ = 1200 mm) and those of the reference specimens (without strengthening).

### 3.3. Failure Mode

**Figure 14:**
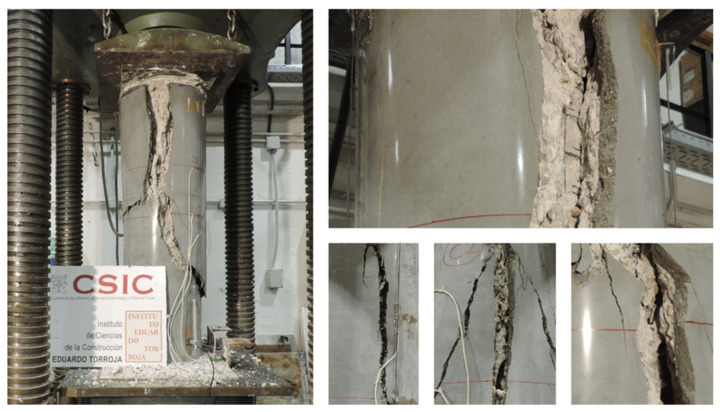
Details of the failure of circular columns.

**Figure 15:**
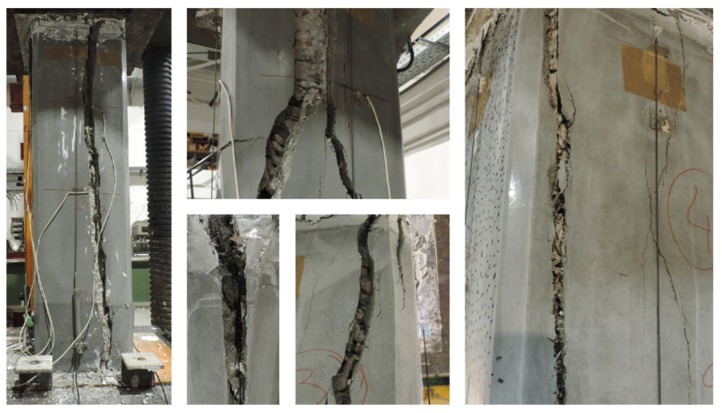
Details of the failure of square columns.

**Figure 16:**
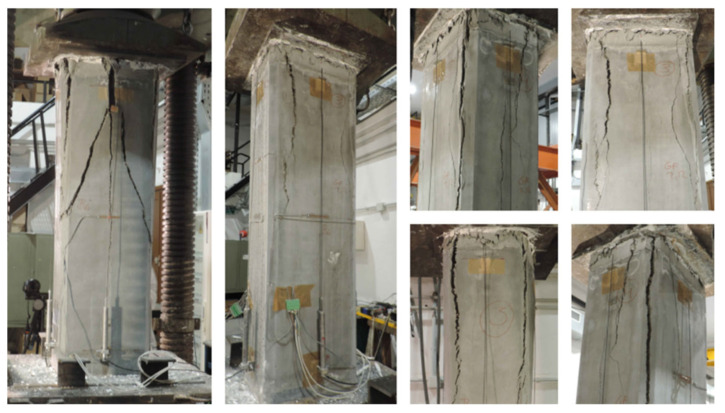
Details of the failure of rectangular columns with b/h = 1.5.

**Figure 17:**
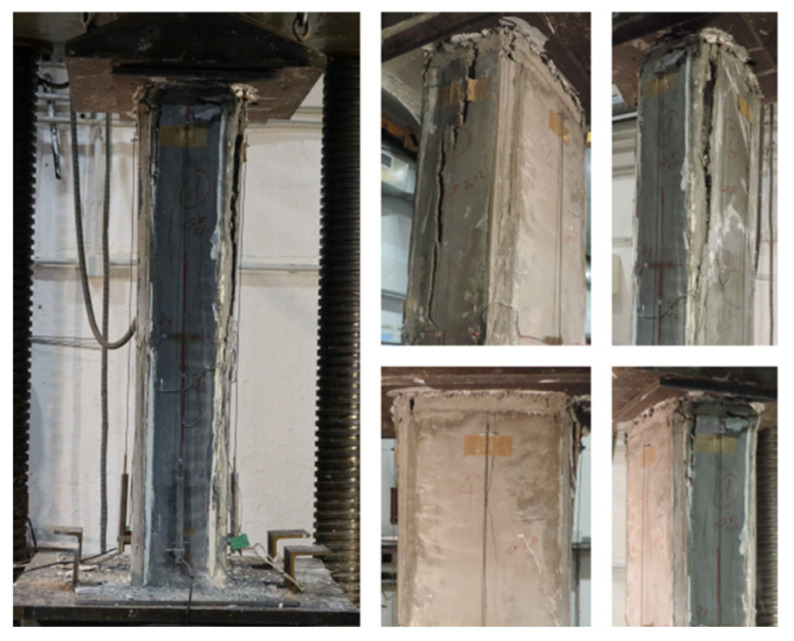
Details of the failure of rectangular columns with b/h = 2.

The failure mode observed in the specimens is analyzed below, particularly those strengthened with HPFRC jacketing. Figure 14 shows photographs of the failure in the strengthened circular columns, while Figure 15, Figure 16 and Figure 17 illustrate the failure of columns with other cross-sectional types.

In the strengthened columns, vertical cracks generally appear at the top of the column and progress downward. Initially, these cracks are narrow and short, allowing the element to continue bearing a load. Toward the end of the test, the cracks widen into openings several centimeters wide, leading to failure.

Unlike other strengthening systems, this study’s specimens reinforced with HPFRC jackets did not fail explosively. Instead, cracking progressed gradually until failure. This behavior is likely influenced by the presence of fibers, which act as a “stitching” mechanism, delaying crack propagation during the initial loading phases.

The typical failure mode of concrete under compression was observed in the reference columns, without external reinforcement. Approximately vertical cracks appeared as the load approached the failure threshold.

## 4. Discussion

To analyze the improvement in axial load-bearing capacity achieved through strengthening, the compressive strength of the concrete in each column at the time of testing (f_c_) was considered. Only by accurately assessing the contribution of the concrete core is it possible to estimate the enhancement provided by the mortar jacketing.

For this reason, the simplified parameters (f′_c_/f_c_) and (P_max_/P_o,theo_), presented in Table 2, were calculated. Although their values differ, they reveal a consistent trend, which is discussed in the following analysis.

Nevertheless, the parameter (P_max_/P_o,theo_) can be considered more representative, as it compares the maximum load obtained in the test with that of an unstrengthened column having the concrete compressive strength measured at the time of testing.

### 4.1. Analysis of the Jacket Height Variable

This variable was studied only in specimens with circular and square cross-sections, as the results allowed conclusions to be drawn without testing other types of section.

In specimen C1, where the strengthening did not extend over the full height of the column, no significant improvement was observed compared to the reference specimen (C0), with only a 9% increase in the (P_max_/P_o,theo_) ratio. In contrast, specimen C2, which was fully jacketed along its entire height, exhibited a substantially higher increase of 67%.

A similar behavior was observed in the square specimens. Specimen S1, which had partial jacketing, exhibited a limited improvement of 43% in the (P_max_/P_o,theo_) ratio, compared to specimens S2 and S3, which were fully jacketed and showed improvements of 105% and 87%, respectively.

Although interrupting the jacketing a few centimeters below the top slab facilitates the implementation, the tests indicate that, in such cases, the strengthening does not significantly improve the axial load-bearing capacity. Therefore, this practice is not recommended.

### 4.2. Mechanical Behavior of Strengthened Columns

The load–strain curves (Figure 13) show the mechanical behavior exhibited by the strengthened specimens during the test.

The full-height jacketed columns generally exhibit greater stiffness than the unstrengthened specimens, with linear behavior up to approximately half the ultimate load. The axial strain obtained for the maximum load (ε_c,axial_) is around 0.18%, a typical value for concrete. The lateral strains recorded for the maximum load are not high, not exceeding 0.05% in the full-height jacketed columns. No plastic phase is observed in the load-strain curve: the strengthened column fails once the maximum load is reached.

This more rigid mechanical behavior coincides with that reported in experimental campaigns by other authors [29] and results in a moderate increase in ductility. However, the literature on the subject indicates that HPFRC jacketing also enhances the axial deformation capacity, leading to a greater increase in ductility. The observed difference may be attributed to the parameters in the other studies, such as smaller specimen sizes or a higher ratio of jacket thickness to the main dimension of the external cross-section. This helps the jacket to confine the inner concrete core. For example, in Xie et al. [27], the samples are made of mass concrete with 300 mm high and 150 mm wide and a jacket thickness up to 40 mm.

It should be noted that in the study carried out, it was observed that the full-height jacketed columns did not fail explosively, but rather after a process of progressive cracking, which may constitute a form of warning of failure.

### 4.3. Analysis of the Impact of Varying the Cross-Sectional Shape of the Column

In this section, the analysis focuses on specimens where the jacket extends over the entire height of the column (h_m_ = 1200 mm), enabling it to perform effectively.

In circular test specimen C2, an increase in axial load-bearing capacity is observed: by 27% in the ratio (f′_c_/f_c_) and 67% in (P_max_/P_o,theo_). The square-section columns (S2 and S3) showed more significant improvements: 43% and 31% in (f′_c_/f_c_), and 105% and 87% in (P_max_/P_o,theo_).

Rectangular columns with a b/a ratio of 1.5 (R1 and R2) also improved their axial load-bearing capacity: 34% and 27% in (f′_c_/f_c_), and 87% and 77% in (P_max_/P_o,theo_). The rectangular column with a b/a ratio of 2 (XR1) achieved gains of 34% in (f′_c_/f_c_) and 83% in (P_max_/P_o,theo_). As mentioned above, the parameter (P_max_/P_o,theo_) can be considered more representative of the degree of improvement obtained.

**Figure 18:**
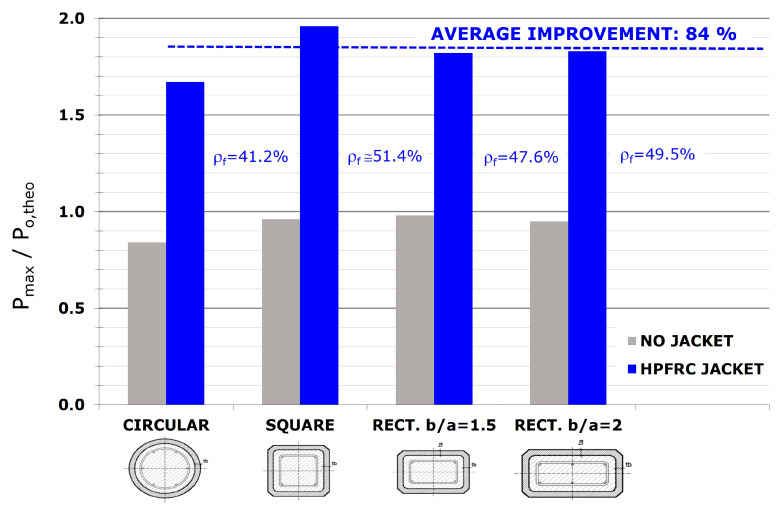
Improvement in load-bearing capacity for different cross-sectional shapes.

Figure 13 shows how, regardless of the shape of the section, the mechanical behavior has been very similar in the fully jacketed columns. Figure 18 illustrates the enhancement in the (P_max_/P_o,theo_) ratio obtained for the different shapes of the cross-section. The differences observed between the various cross-sectional configurations are relatively small. Any variations that do occur could be associated with the geometric strengthening ratio (ρ_m_) used in each case (also indicated in the figure). This issue is addressed in the following subsection.

**Figure 19:**
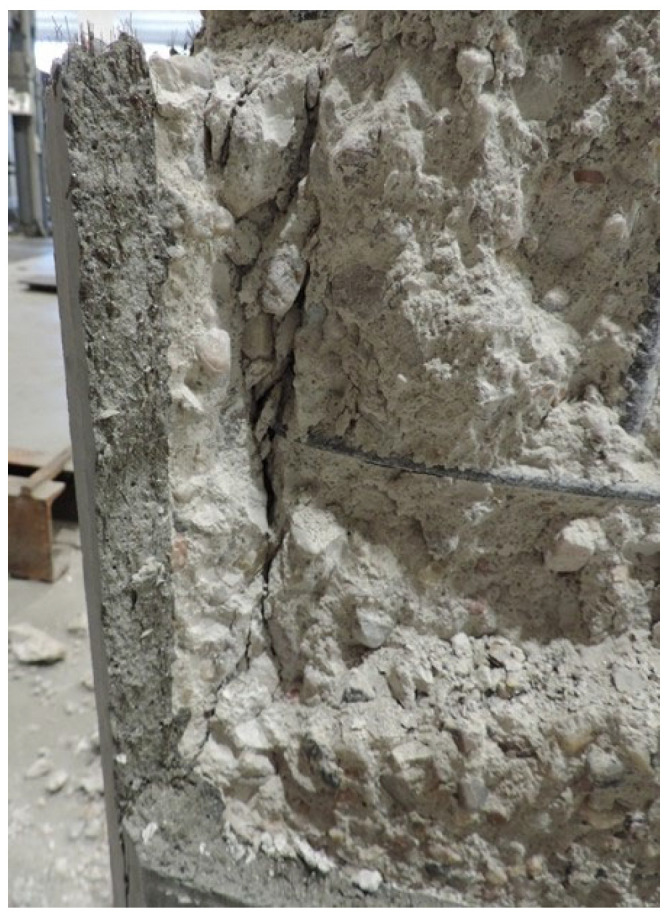
Column tested—good adhesion of the jacket to the substrate.

Simple sandblasting ensures good adhesion between the jacket and the substrate (Figure 19). No failures due to lack of adhesion were observed until the end of the test, when the crack widened by several centimeters and led to complete failure.

### 4.4. Analysis of the Impact of Varying the Geometric Strengthening Ratio

Table 1 shows each test specimen’s geometric strengthening ratio (ρ_m_). As indicated, the aim was for the columns to have a similar gross area of concrete, though not identical. While the thicknesses of the jackets are comparable, variations arise due to the reduction in sectional dimensions after sandblasting and the standardized dimensions of the commercial external formwork. Consequently, the geometric strengthening ratios of the columns are similar, but not identical.

**Figure 20:**
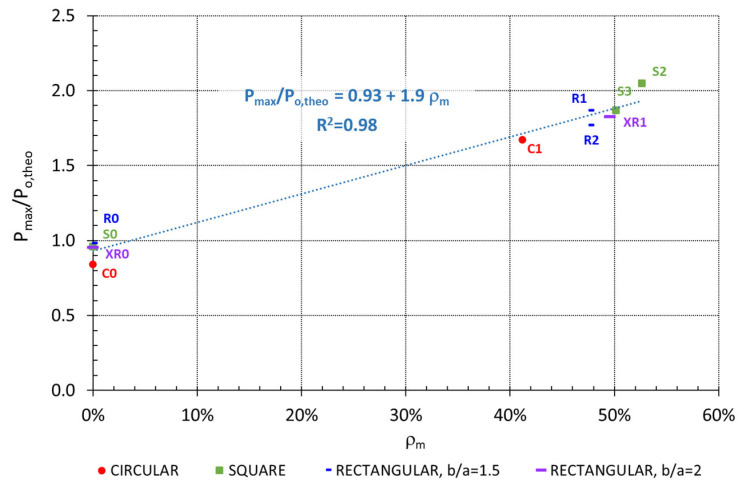
Improvement in load-bearing capacity versus geometric strengthening ratio.

Figure 20 illustrates the improvement in axial load capacity relative to the geometric strengthening ratio (ρ_m_). As shown, the influence of this parameter has been significant, and a linear trend can even be identified.

### 4.5. Analysis of the Mechanism of Structural Strengthening

The experimental results suggest that, under the conditions of this experimental study (type of mortar and steel fiber content, ratio of jacket thickness to concrete core and specimen dimensions more representative of real columns), the HPFRC jacket would not have acted as confinement reinforcement.

In general, for confinement to occur in a compressed concrete element, two conditions must be satisfied: (1) a significant increase in compressive strength and (2) a substantial enhancement in ultimate strain.

The tests showed a substantial increase in load-bearing capacity when the jacket covered the full height, with an average (P_o_/P_max_) improvement of 84% (Table 2). However, the ultimate strain remained close to that of unconfined concrete, indicating that the second confinement condition was not satisfied.

The observed improvement can be attributed to the enlargement of the cross-sectional area through the addition of the HPFRC jacket, which possesses a high compressive strength. As a result, the shape of the cross-section (circular, square, or rectangular with varying proportions) did not significantly affect the degree of improvement.

This could be particularly advantageous for strengthening non-circular columns, which are common in buildings. Unlike FRP confinement, whose effectiveness decreases in rectangular columns with aspect ratios above 1.5 [47] and which requires protective layers against impact or fire, HPFRC jacketing is not subject to these limitations.

## 5. Conclusions

This paper presents the experimental results of twelve RC columns, as part of a study on HPFRC jacketing applied to building columns, to improve their axial compression capacity with minimal intervention.

The columns are 1200 mm high, made of concrete with a 25–30 MPa compressive strength, and reinforced longitudinally and transversely with B500S steel. They are externally strengthened with a high-strength concrete jacket reinforced with steel fibers (HPFRC). The jacket is thin (less than 30 mm) and has no additional internal steel reinforcement. The main parameters of the study are as follows: (1) the shape of the column cross-section (circular, square and rectangular with different ratios between the lengths of the sides) and (2) the jacket height (covering the entire height of the column or leaving the last 50 mm free).

The main conclusions from the study are as follows:

- The application of a thin HPFRC jacket along the full height of RC columns led to a significant improvement in axial compressive capacity. Experimental results revealed a load-bearing capacity enhancement between 67% and 105%, with an average improvement of 84%, when compared to an unreinforced column of equivalent concrete strength.
- Partial HPFRC jacketing, applied without covering the full height of the column, resulted in notably lower gains (between 9 and 43%), indicating it is not recommended.
- The cross-sectional shape did not affect the efficacy of the fully jacketed columns tested, with maximum gains ranging from 87% to 105% for square columns and 77% to 87% for rectangular ones.
- The geometric strengthening ratio (ρₘ = A_n_/A_m_) is a key parameter for HPFRC jacketing. The best results were achieved in fully jacketed columns with higher geometric ratios, regardless of the cross-sectional shape. One square column with the largest strengthening ratio (ρₘ = 52.6%) doubled the load-bearing capacity.
- Fully jacketed columns showed increased stiffness and a nearly linear response up to about 50% of the maximum load. Despite moderate improvement in ductility, they failed progressively rather than explosively, providing a warning before collapse.
- The HPFRC jacket did not significantly confine the concrete core, as evidenced by the substantial increase in load-bearing capacity when the jacket covered the entire height, while the ultimate strain remained similar to that of unconfined concrete. Instead, the improvement primarily resulted from the increased cross-sectional area.

These findings indicate that thin HPFRC jacketing can be a versatile solution offering practical advantages over systems like FRP, particularly for rectangular RC columns.

Further experimental research is needed to confirm these results. These conclusions should be viewed in the context of the specific parameters studied and the limitations related to the number of tests available.

## Figures and Tables

**Table 1:** Main parameters of specimens.

Column	H [mm]	h_m_ [mm]	b * [mm]	a * [mm]	t_b_ [mm]	t_a_ [mm]	f_c_ [mm]	A_s_ [mm]	A_n_ [mm]	A_m_ [mm]	ρ_m_ [%]	t/b_ext_
C0	1200	-	300	-	-	-	24.1	678.6	70,007.2	0.0	-	-
C1_h1150	1200	1150	295	-	27.5	-	25.4	678.6	67,670.7	27,862.0	41.2	0.08
C2_h1200	1200	1200	295	-	27.5	-	26.7	678.6	67,670.7	27,862.0	41.2	0.08
S0	1200	-	250	-	-	-	35.0	452.4	61,704.3	0.0	-	-
S1_h1150	1200	1150	245	-	28.5	-	24.1	452.4	59,229.3	31,179.0	52.6	0.09
S2_h1200	1200	1200	245	-	28.5	-	27.4	452.4	59,229.3	31,179.0	52.6	0.09
S3_h1200	1200	1200	247	-	27.5	-	28.4	452.4	60,213.3	30,195.0	50.1	0.09
R0_ref	1200	-	300	200	-	-	25.2	452.4	59,204.3	0.0	-	-
R1_h1200	1200	1200	295	195	25.0	25.0	28.1	452.4	56,729.3	27,000.0	47.6	0.07
R2_h1200	1200	1200	295	195	25.0	25.0	32.2	452.4	56,729.3	27,000.0	47.6	0.07
XR0_ref	1200	-	350	175	-	-	28.1	678.6	60,228.1	0.0	-	-
XR1_h1200	1200	1200	345	170	27.5	24.0	26.3	678.6	57,628.1	28,550.0	49.5	0.07

* Real dimensions measured after sandblasting.

**Table 2:** Main results obtained.

Column	P_max_ [kN]	ε_c,axial_ [%]	ε_c,lat_ [%]	f′_c_ [MPa]	f’_c_/f_c_		P_o,theo_ [kN]	P_max_/P_o,theo_	
C0	1649.1	0.13	0.01	21.1	0.87		1960.01	0.84	
C1_h1150	2163.8	0.11	0.01 *	21.2	0.83		1990.27	1.09	
C2_h1200	3473.5	0.17	0.02	34.0	1.27		2078.24	1.67	
S0	2247.0	0.18	0.04	33.8	0.97		2337.52	0.96	
S1_h1150	2293.8	0.08	0.02 *	24.5	1.02		1605.42	1.43	
S2_h1200	3690.9	0.17	0.02 *	39.2	1.43	M: 1.37 SD: 0.08 CV: 6.05%	1800.88	2.05	M: 1.96 SD: 0.13 CV: 6.46%
S3_h1200	3531.2	0.18	0.05	37.3	1.31		1888.00	1.87	
R0_ref	1641.80	0.14	0.02	25.52	1.01		1675.27	0.98	
R1_h1200	3307.50	0.18	0.04	37.57	1.34	M: 1.30 SD: 0.05 CV: 3.80%	1772.21	1.87	M: 1.82 SD: 0.07 CV: 3.79%
R2_h1200	3555.20	0.14	0.05	40.92	1.27		2009.91	1.77	
XR0_ref	1868.00	0.18	0.04	26.90	0.96		1960.83	0.95	
XR1_h1200	3259.00	0.17	0.04 *	35.15	1.34		1784.75	1.83	
Average partially jacketed columns (h_m_ = 1150 mm)					0.93				1.26
Average fully jacketed columns (h_m_ = 1200 mm)					1.33				1.84

* In these cases, the gauge readings were lost before reaching maximum load, so the maximum strain recorded before losing these readings is collected.

## Data Availability

The original contributions presented in this study are included in the article. Further inquiries can be directed to the corresponding author.
